# Communicating Absolute Fracture Risk Reduction and the Acceptance of Treatment for Osteoporosis

**DOI:** 10.1007/s00223-022-00948-2

**Published:** 2022-02-13

**Authors:** Katherine A. P. Ralston, Jonathan Phillips, Amrey Krause, Barbara Hauser, Stuart H. Ralston

**Affiliations:** 1 Medicine for the Elderly, NHS Lothian, Western General Hospital, Edinburgh, EH4 2XU UK; 2 Centre for Genomic and Experimental Medicine, MRC Institute of Genetics and Cancer, Western General Hospital, University of Edinburgh, Edinburgh, EH4 2XU UK; 3 Edinburgh Parallel Computing Centre, University of Edinburgh, Edinburgh, UK; 4 Rheumatic Diseases Unit, NHS Lothian, Western General Hospital, Edinburgh, EH4 2XU UK

**Keywords:** Osteoporosis, Bisphosphonates, Decision aids, Fracture risk

## Abstract

Healthcare professionals frequently communicate the benefits of treatments as a relative risk reduction (RRR) in the likelihood of an event occurring. Here we evaluated whether presenting the benefits of osteoporosis treatment as a RRR in fractures compared with an absolute risk reduction (ARR) changed the patient’s attitudes towards accepting treatment. We surveyed 160 individuals attending a specialised osteoporosis clinic for face-to-face consultations between May 2018 and Jan 2021. They were presented with information on RRR for the treatment being considered followed by ARR and after each question were asked about how likely they would be to start treatment on a 5-point scale (1 = very likely, 5 = very unlikely). Participants were less likely to accept treatment when it was presented as ARR (mean score 2.02 *vs.* 2.67, *p* < 0.001, 95% CI for difference − 0.82 vs − 0.47) and thirty-eight participants (23.7%) declined treatment with knowledge of their ARR when they would have accepted the same treatment based on the RRR. Individuals who declined treatment had a lower 5-year risk of fracture than those who accepted treatment (9.0 vs. 12.5%, *p* < 0.001, 95% CI − 5.0 to − 1.6) and as fracture risk decreased, the participant was less likely to accept treatment (Spearman *r* − 0.32, 95% CI − 0.46 to − 0.17, *p* ≤ 0.001). Whilst presentation of data as ARR more accurately reflects individual benefit and helps facilitate shared decision-making, clinicians should be aware that this will lead to a proportion of patients with lower fracture risk declining treatment for osteoporosis.

## Introduction

Osteoporosis is a common condition characterised by low bone mass and deterioration in bone architecture, leading to bone fragility and increased fracture risk [1]. A variety of treatments are now available which have been shown to reduce the risk of fractures in patients with osteoporosis and osteopenia, although none can completely prevent the occurrence of fractures [2]. Whilst these treatments are quite effective at preventing hip and vertebral fractures with a 40% reduction for hip fractures as compared with placebo and a 50–75% reduction for vertebral fractures, efficacy for the prevention of non-vertebral, non-hip fractures, which are the most common fracture types is limited with a reduction in risk of only 15–20% compared with placebo which means that the benefits in terms of fracture prevention for many patients is modest [3].

When discussing the risks and benefits of treatment with patients, healthcare professionals frequently communicate the potential benefits of treatment in terms of a relative risk reduction (RRR) in an event, and previous research has indicated that this increases the likelihood of people accepting treatment [4]. It has also been pointed out that presentation of data as RRR is often misleading [5]. For example, in the context of osteoporosis, many bisphosphonates reduce the relative risk of hip fracture by 40% compared with placebo. Although this sounds impressive, the absolute benefit in terms of hip fractures prevented in osteopenic women with the characteristics of those treated by Reid and colleagues [6] corresponds to a reduction from 12 fractures per 1000 women treated for 6 years to 8 fractures. This equates to an absolute reduction of 0.4%, 100 times less than the RRR.

Decision aids can help patients understand the potential benefits of different treatment options, including the option of no treatment, and improve both knowledge about the condition and clarity about what matters to them [7, 8]. This is particularly relevant when considering the treatment of osteoporosis, where the aim of treatment is to prevent a fracture occurring rather than to help symptoms. Here we compared patients’ attitudes to accepting osteoporosis treatments when the benefits of treatment were presented in terms of absolute risk reduction (ARR) as opposed to RRR.

## Patients and Methods

This was an observational single centre study of patients with osteoporosis who were referred for face-to-face clinic consultations at the osteoporosis clinic at NHS Lothian between 7th May 2018 and 5th Jan 2021. The aim of the study was to gather information on at least 150 individuals, but no formal power calculation was made to decide upon the sample size.

Patients were included if treatment for osteoporosis was being considered because of low BMD on DXA or low trauma vertebral fractures according to the Scottish Intercollegiate Guidelines Network (SIGN) 142 guideline [9]. The type of treatment being discussed was based on normal clinical practice in our locality where oral bisphosphonates are the first choice unless there is a contraindication. Intravenous zoledronic acid is used where there is a contraindication to oral bisphosphonates or where patients have had intolerance to oral bisphosphonates. The anabolic treatments teriparatide and romosozumab are offered to individuals with severe spinal osteoporosis with vertebral fractures, largely based on the results of the VERO [10] and ARCH [11] studies which have shown anabolic treatments to be superior to oral bisphosphonates in these circumstances. Denosumab is a further treatment option but is used relatively infrequently in view of the risk of rebound high bone turnover and vertebral fractures on discontinuation [12].

The only exclusion criterion was if the patient was already on osteoporosis treatment at the time of consultation. The risk of fracture for individual patients was calculated using the FRAX calculator over a 5-year time frame using major osteoporotic fracture as the metric to present to the patient. A 5-year timeframe was used since this is has previously been suggested as an appropriate timeframe for the initial duration of treatment [13, 14]. In the case of bisphosphonates, the Medicines and Healthcare products Regulatory Agency (MHRA) in the UK recommends that the need for ongoing therapy is reviewed after 5 years of use as a risk minimization measure to prevent adverse events associated with long term suppression of bone turnover such as atypical femoral fractures and osteonecrosis of the jaw.

For anabolic drugs like teriparatide and romosozumab we assumed that the reduction in risk of fracture during treatment would be sustained for 5 years as the result of follow-on treatment with antiresorptive medication. Although this has not yet been proven for romosozumab, observational studies suggest that the beneficial effects of teriparatide are sustained following introduction of antiresorptive therapy for at least 5 years [15].

An estimate of the ARR for the patient was reached by calculating the RRR for the treatment being considered based on the network meta-analysis published by Barrionuevo [2] considering the participants absolute fracture risk.

As part of the consultation in which the risks and benefits of the treatment being considered was discussed, participants were asked by the attending clinician what the likelihood was that they would be willing to accepting treatment over a 5-year interval when presented with information on how effective the treatment would be at preventing a future fracture expressed as a RRR on a five-point scale (very likely, likely, maybe, unlikely, very unlikely). The participant was then asked whether their intention would be to accept treatment or not. Next, participants were asked to rate their willingness to accept treatment when the data were presented as an ARR. They were again asked whether their intention would be to accept treatment or not. If the participants decided not to accept treatment, this was noted and recorded as a change in the final decision.

The data were analysed by SPSS statistics version 25. A code was assigned to the five-point scale (1 = very likely, 2 = likely, 3 = maybe, 4 = unlikely, 5 = very unlikely) for the dataset.

Since the data were not normally distributed a Wilcoxon paired rank test was used to assess differences between codes when presented with information on relative and ARR. Spearman non-parametric correlation test was used to assess the correlation between absolute fracture risk and likelihood of accepting treatment. Binary logistic regression analysis was used to identify predictors of patients declining treatment after having been presented with information on ARR. For this analysis, sex, age, absolute fracture risk without treatment, type of treatment being considered (oral antiresorptive, parenteral antiresorptive and parenteral anabolic), the presence of low trauma vertebral fractures and the Scottish Index of Multiple Deprivation (SIMD) were entered into the model based on the patients’ postcode. The SIMD looks at the extent to which an area is deprived across seven domains: income, employment, education, health, access to services, crime, and housing (www.simd.scot).

## Results

We studied 160 participants, of whom 150 were female. Their mean ± SD age was 73.1 ± 10.1 years. The treatments under consideration were oral alendronic acid (*n* = 104, 65%), oral risedronate (*n* = 5, 3.1%), intravenous zoledronic acid (*n* = 27, 16.9%), teriparatide (*n* = 21, 13.1%) romosozumab (*n* = 2, 1.3%) and denosumab (*n* = 1, 0.6%). There was a history of low trauma vertebral fractures in 99 patients (61.9%) and low trauma non-vertebral fractures in 79 individuals (49.4%); 26 (16.3%) individuals had low BMD values (*T* score ≤ − 2.5 at spine or hip) without a previous history of fracture.

**Fig. 1:**
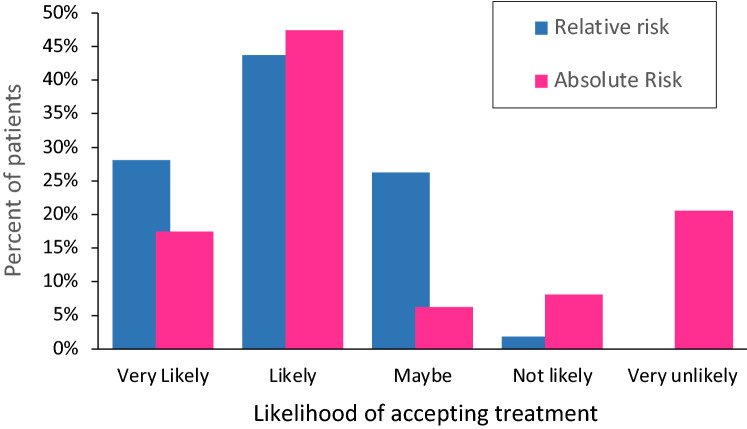
Attitudes to accepting treatment for osteoporosis according to presentation of benefits as absolute or relative risk reduction. The proportion of patients who said they would accept treatment when the benefits were presented in terms of a relative risk reduction (blue columns) compared with an absolute risk reduction (red columns). The difference between groups was highly significant (*p* < 0.001)

The participants likelihood of accepting treatment was significantly lower when data on the likely benefits were presented as ARR versus RRR. Accordingly, the mean score on the five-point scale was: ARR 2.67 (95% CI 2.45 to 2.89) vs. RRR 2.02 (95% CI 1.89 to 2.14), *p* ≤ 0.001). (Fig. 1). In total, 38 participants (23.8%) decided not to go ahead with anti-osteoporosis treatment when the benefits were expressed as ARR compared with RRR. There was a significant correlation between likelihood of accepting treatment and 5-year fracture risk overall. As fracture risk decreased, the participant was less likely to accept treatment (Spearman *r* − 0.32, 95% CI − 0.46 to − 0.17, *p* ≤ 0.001). Participants who changed their mind and declined treatment had a significantly lower 5-year risk of fracture than those who did not: (9.0% (95% CI 8.2% to 10.5%) vs. 12.5% (95% CI 11.6% to 13.7%), *p* ≤ 0.001).

To identify other potential predictors of patients changing their mind about accepting treatment when presented with information on ARR, a binary logistic regression analysis was performed entering age, sex, type of osteoporosis treatment, presence of previous low trauma vertebral fractures, and social deprivation index as explanatory variables. This showed that absolute fracture risk without treatment was the most significant predictor (*β*-statistic − 0.233, standard error 0.06, *p* < 0.0001) followed by age (*β*-statistic + 0.061, standard error 0.024, *p* = 0.011). None of the other variables was identified as a significant predictor of the patient changing their mind about treatment. This indicates that older patients with a low absolute fracture risk were most likely to decline treatment after being presented with information on RRR followed by ARR.

## Discussion

This study has shown that when information on the potential benefits of anti-osteoporosis treatment is presented in terms of absolute benefit as opposed to relative benefit, participants were significantly less inclined to accept treatment and 23.7% declined the offer of treatment having previously indicated that they would go ahead with treatment. There was a significant correlation between absolute fracture risk without treatment and participants likelihood of accepting treatment. In keeping with this, participants who declined treatment had a significantly lower fracture risk than those who accepted. Furthermore, logistic regression analysis showed that a low absolute fracture risk without treatment was the most important predictor of the participant changing their mind about having treatment followed by increasing age. This indicates that older patients with a low absolute fracture risk were most likely to decline treatment after being presented with information on RRR followed by ARR. It was not possible to identify a specific cutoff for either age or baseline fracture risk which would predict that a patient may decline treatment after being presented with information on ARR.

These findings are in line with previous work in other fields of medicine which has shown that the way in which risk is presented can have a major factor in influencing decisions [4]. Framing risk in absolute terms leads to a more accurate view of risks and benefits, and an increase in ‘normative’ decision-making [16]. However, unlike many previous studies in this field which are based on hypothetical situations, we assessed participants who were responding to information given to them on treatments being offered in routine clinical practice. This gives us an insight into the ‘real-world’ effect of the importance of accurate risk communication with patients. Healthcare professionals have a duty to ensure patients are aware of benefits and risks of treatment so that they can make an informed decision [17]. Both patients and clinicians tend to over-estimate benefits and under-estimate harms of treatments [18, 19]. This is particularly true when RRRs are used to present the benefits of treatment [16, 20, 21].

The study has some weaknesses; it was based in a secondary care osteoporosis service, dealing with patients who may have had more severe or complicated osteoporosis than is dealt with in primary care. It would be interesting to extend this study to include patients being considered for treatment in a primary care setting.

Whilst it is important that patients are empowered to make informed choices on their care, it is also relevant to consider the implications of this decision on a population level. For example, a small reduction in absolute risk of fracture for an individual may become significant on a population level in terms of reducing incidence of fracture and costs to healthcare services overall. It would be interesting to conduct further studies on the impact which this might have made on individual decision-making.

Our observations indicate that presentation of benefits of osteoporosis treatment in terms of ARR impacts on the patient’s attitude to accepting treatment. In order to facilitate discussion on the benefits of treatment we have developed the “Osteoporosis Risk Benefit Calculator” (ORB) (available from App store and Google play and also online at: https://webapps.igmm.ed.ac.uk/world/research/rheumatological/ORBCalculator/).

This calculator allows healthcare professionals and patients to assess absolute benefit of different treatments by entering information on fracture risk and selecting different treatments. The information can be viewed numerically in terms of the likelihood of having different types of fracture with and without treatment over the time interval being discussed or using a visual aid in which the total number of fractures without and with treatment is displayed. The advantage of using the calculator is that it allows both clinicians and patients to look at the potential benefits of different treatments that are being discussed. We believe that this should be a useful decision aid for patients and clinicians alike when treatment for osteoporosis is being considered.

## Patient and Public Involvement

The concept for the study came from informal discussions with patients when the need for an easy-to-use calculator to display the benefits of different treatments was highlighted as being desirable by lay members of the SIGN 142 guideline group.
